# L-arginine and N-carbamoylglutamic acid supplementation enhance young rabbit growth and immunity by regulating intestinal microbial community

**DOI:** 10.5713/ajas.18.0984

**Published:** 2019-05-28

**Authors:** Xiaoming Sun, Jinglin Shen, Chang Liu, Sheng Li, Yanxia Peng, Chengzhen Chen, Bao Yuan, Yan Gao, Xianmei Meng, Hao Jiang, Jiabao Zhang

**Affiliations:** 1 Laboratory Animal Center, College of Animal Science, Jilin University, Changchun, Jilin, 130062, China; 2 School of Grains, Jilin Business and Technology College, Changchun, Jilin, 130507, China

**Keywords:** L-arginine, N-carbamoylglutamic Acid, Japanese White Rabbits, Intestinal Development, Intestinal Microbial Community

## Abstract

**Objective:**

An experiment was conducted to determine the effects of L-arginine (L-Arg) and N-carbamoylglutamic acid (NCG) on the growth, metabolism, immunity and community of cecal bacterial flora of weanling and young rabbits.

**Methods:**

Eighteen normal-grade male weanling Japanese White rabbits (JWR) were selected and randomly divided into 6 groups with or without L-Arg and NCG supplementation. The whole feeding process was divided into weanling stage (day 37 to 65) and young stage (day 66 to 85). The effects of L-Arg and NCG on the growth, metabolism, immunity and development of the ileum and jejunum were compared via nutrient metabolism experiments and histological assessment. The different communities of cecal bacterial flora affected by L-Arg and NCG were assessed using high-throughput sequencing technology and bioinformatics analysis.

**Results:**

The addition of L-Arg and NCG enhanced the growth of weanling and young rabbit by increasing the nitrogen metabolism, protein efficiency ratio, and biological value, as well as feed intake and daily weight gain. Both L-Arg and NCG increased the concentration of immunoglobulin A (IgA), IgM, and IgG. NCG was superior to L-Arg in promoting intestinal villus development by increasing villus height, villus height/crypt depth index, and reducing the crypt depth. The effects of L-Arg and NCG on the cecal bacterial flora were mainly concentrated in different genera, including *Parabacteroides*, *Roseburia, dgA-11_gut_group*, *Alistipes*, *Bacteroides*, and *Ruminococcaceae_UCG-005*. These bacteria function mainly in amino acid transport and metabolism, energy production and conversion, lipid transport and metabolism, recombination and repair, cell cycle control, cell division, and cell motility.

**Conclusion:**

L-Arg and NCG can promote the growth and immunity of weanling and young JWR, as well as effecting the jejunum and ileum villi. L-Arg and NCG have different effects in the promotion of nutrient utilization, relieving inflammation and enhancing adaptability through regulating microbial community.

## INTRODUCTION

The rabbit is an herbivore. Its cecum is well developed and contains approximately 10^10^ to 10^12^ microorganisms per gram. The community structure in the flora plays a critically important role in the digestion of feed, nutrient absorption and immune organ development [1]. Intestinal microflora of rabbits in different growth stages varies; a stable community structure is formed from an almost aseptic state and maintains a dynamic balance to adapt to the animal’s feeding habits [2]. However, this dynamic balance is easily broken, which affects the physiology and growth state of the rabbit’s intestines and can even affect the entire individual. This imbalance may promote the growth of rabbits, but may also inhibit growth and even result in death [3]. Therefore, the structure and corresponding physiological functions of the intestinal microflora of rabbits have become increasingly studied.

The traditional research methods for investigating intestinal bacterial communities mainly involve selective culture of microorganisms, which are identified by typing. This method has low accuracy, is time-consuming and entails a heavy workload [4]. Moreover, less than 10% of bacteria in the intestinal microflora are culturable [5], so it is difficult to study the complex intestinal microbial community deeply. In recent years, high-throughput sequencing technology has been widely used to analyze the diversity and abundance of intestinal microbial communities, as it offers real-time detection and significant quantitative advantages [6], to improve our understanding of the composition and function of the intestinal microbial community.

As an essential amino acid for young animals, arginine (Arg, usually provided via L-arginine as a feed additive) and its precursor substance N-carbamoylglutamic acid (NCG) also play an important role in animal growth. As feed additives, appropriate amounts of L-Arg can promote feed intake, daily weight gain, feed utilization rate, relieve oxidative stress, prevent intestinal atrophy, promote proliferation and differentiation of intestinal villus cells, and reduce apoptosis in juvenile animals. NCG can also improve animal reproductive performance and further increase feed intake daily gains and feed-to-weight ratios [7]. However, studies regarding whether L-Arg and NCG change the structure of the intestinal flora of rabbits are still limited.

In this study, L-Arg and NCG were added to basal rabbit diets, and high-throughput sequencing technology was used to determine how the structure and abundance of cecal flora in rabbits changed over time. Our results provide a scientific foundation for the effects of L-Arg and NCG and provide new understanding for the role of animal organisms and effective regulation of the overall metabolic level of animals.

## MATERIALS AND METHODS

### Experimental design

In this study, 18 normal-grade male weanling Japanese White rabbits (JWR) were selected and randomly divided into 6 groups. Two groups served as controls, and 4 groups represented various treatment groups, three of which were used as biological replicates. The grouping scheme is shown in Supplementary Figure S1. The control group was the basal diet (S1C and S2C groups), and the treatment group diets were supplemented with 0.5% L-Arg (S1L and S2L groups) and 0.5% NCG (S1N and S2N groups) in the basal diet. The composition and nutritional levels of the diets are shown in Table 1. The whole feeding process was divided into two stages: weanling rabbits (Stage 1, S1, day 37 to 65) and young rabbits (Stage 2, S2, day 66 to 85). All rabbits were ultimately euthanized; fecal, urine and blood samples were collected at 65 and 85 days of age, and additional samples were collected and the remaining indicators were assessed. All experiments were conducted at the Experimental Animal Center of Jilin University in accordance with the Institutional Animal Care and Use Committee (IACUC ID: 201712003) of Jilin University.

### Sample collection

All rabbits were housed in independent metabolic cages and provided food and water *ad libitum*. The daily intake, fecal output and urine output of each rabbit were recorded for analysis of growth performance. When the rabbits were 63 to 65 days old and 83 to 85 days old, the feces and urine were collected throughout the day. The samples were collected every day and repeated as an independent experiment from day 63 to 65. After fixing with 10% sulfuric acid, the nitrogen level was assessed by the Kjeldahl method [8].

Ten milliliters of whole blood was collected by cardiac blood sampling, and serum was separated after letting the samples stand for 60 min. Immunoglobulin A (IgA), IgM, and IgG levels were determined by enzyme-linked immunosorbent assay. Subsequently, each collected spleen and thymus without fat and connective tissue were weighed, and an immune organ index was determined. The ileum and jejunum were washed with physiological saline and fixed in a 4% paraformaldehyde solution for the preparation of histochemical sections. Finally, the contents were collected from the cecum and quickly transferred to −80°C until the extraction of bacterial genomic DNA.

### Histological assessment

**Figure 1:**
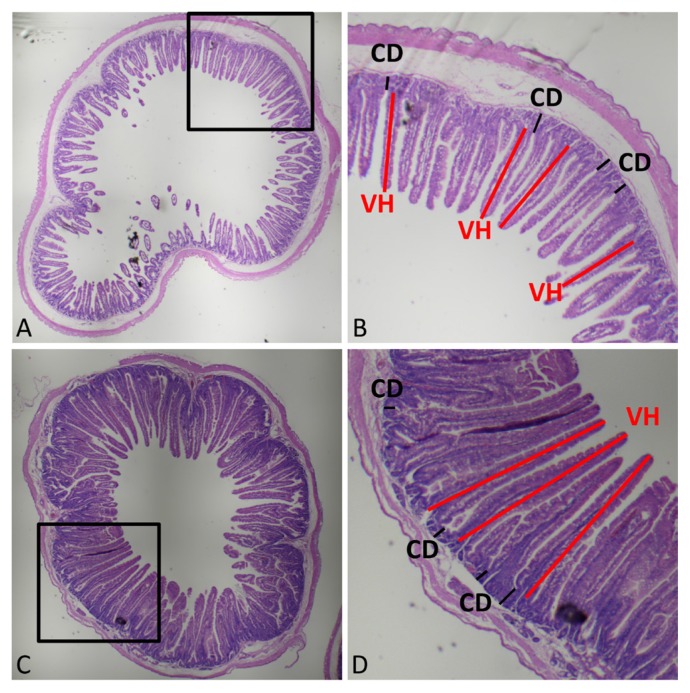
Histomorphological evaluation of ileal and jejunal tissues. Morphological evaluation of small intestinal villi was performed with hematoxylin and eosin (H&E) staining. (A) and (C) represent morphology of the ileum and jejunum, respectively. (B) and (D) represent the enlarged areas of (A) and (C), respectively. VH, villus height; CD, crypt depth. Three fields of fluff integrity and straightness with 10 complete fluffs (length of VH and CD) were selected randomly and measured in each slice.

Morphological evaluation of small intestinal villi was performed on ileal and jejunal sections with hematoxylin and eosin staining. Three fields of fluff integrity and straightness with 10 complete fluffs were selected and measured in each slice. The vertical distance is referred to as the villus height (VH) and the crypt depth (the vertical distance from the crypt opening to the base of the crypt, CD), and the ratio of VH to CD was calculated. The measurement position is shown in Figure 1.

### DNA extraction

Genomic DNA was extracted from 250 mg cecal contents of each rabbit using a PowerSoil DNA Isolation kit (Anbiosci Tech Ltd., Shenzhen, China) according to the manufacturer’s instructions and stored at −80°C until further analysis.

### Polymerase chain reaction amplification and high-throughput sequencing

The V3+V4 region of the 16S rDNA gene was amplified with the forward primer 5′-ACTCCTACGGGAGGCAGCA-3′ and the reverse primer GGACTACHVGGGTWTCTAAT using a MyCycler Thermal Cycler (Bio-Rad Laboratories, Hercules, CA, USA). Polymerase chain reaction (PCR) was performed in triplicate for each sample of the reaction mixture (15 μL) containing 7.5 μL of Phusion High-Fidelity PCR Master Mix (New England Biolabs (Beijing) LTD., Beijing, China), 1.0 μL of each primer (BGI Tech Solutions Co., Ltd., Hongkong, China) (2 μM), 5 μL of template DNA (1 ng/μL) and 1.5 μL of sterile deionized water. PCR conditions were as follows: initial denaturing step at 95°C for 5 min (1 cycle), followed by 25 cycles of 95°C for 30 s, 50°C for 30 s, 72°C for 40 s, and a final extension of 10 min at 72°C. Subsequently, PCR products for each sample were detected using a 1.0% agarose gel and purified using a GeneJET Gel Extraction Kit (Thermo Scientific, Waltham, MA, USA). Sequencing was performed at the Biomarker Technologies Co., LTD (Beijing, China) with an Illumina HiSeq platform.

### Bioinformatics analyses

The raw tags, clean tags, and effective tags sequences were obtained using FLASH v1.2.7 (http://ccb.jhu.edu/software/FLASH/), Trimmomatic v0.33 (http://www.usadellab.org/cms/index.php?page=trimmomatic), and UCHIME v4.2 (http://drive5.com/uchime). The sequences were deeply analyzed with the QIIME v1.8.0 software package (http://qiime.org/) along with custom Perl scripts to analyze alpha- and beta- diversity. Then, the sequences were filtered by QIIME quality filters in order to obtain pure sequencing data. Operational taxonomic units (OTUs) were selected using a de novo OTU picking protocol with a 97% identity threshold, and then, a representative sequence was picked for each OTU using the Mothur software (https://www.mothur.org/) and Silva database (https://www.arb-silva.de/) to annotate taxonomic information for each representative sequence. Alpha diversity calculation includes six metrics: OTUs, Ace, Chao1, Simpson, Shannon, and Coverage indices. Rarefaction curves, Shannon index, and rank abundance curve were generated based on these metrics.

Then, we compared overall samples between intra group compositions using the unweighted pair-group method with arithmetic mean (UPGMA) and Metastats method. Clusters of orthologous groups of proteins (COG) was used to predict changes in metabolic processes caused by microbial communities between samples and between groups.

### Statistical analysis

One-way analysis of variance was used to analyze differences in cecal microflora of rabbits among groups using SPSS 22.0 software (SPSS Inc., Chicago, IL, USA). Significance was reported at p<0.05.

## RESULTS

### Effects of L-arginine and N-carbamoylglutamic acid on Japanese White rabbits growth performance

As shown in Table 2, between the ages of 37 to 65 days, the additional daily addition of L-Arg and NCG increased the average daily gain of the weanling rabbits from 37.38 g to 39.40 (p<0.01) and 39.53 g (p<0.01), respectively. The average daily feed intake was increased from 100.05 g to 101.14 g (p<0.05) and 101.70 g (p<0.05), respectively. At the age of 66 to 85 days, the addition of L-Arg and NCG increased the average daily gain of young rabbits from 37.16 g to 39.15 g (p<0.01) and 39.68 g (p<0.01), respectively, and the food intake was increased from 125.46 g to 128.75 g (p<0.01) and 131.33 g (p< 0.01), respectively. However, neither stage has a significant difference on the feed conversion rate between the L-Arg- and NCG-supplemented groups.

### Effects of L-arginine and N-carbamoylglutamic acid on Japanese White rabbits nitrogen metabolism

As shown in Table 3, in the weanling rabbit stage, the addition of L-Arg and NCG increased intake nitrogen (IN) from 3.39 g/d to 3.55 g/d and 3.53 g/d (p<0.01) and increased fecal nitrogen (FN) from 0.60 g/d to 0.67 g/d and 0.67 g/d (p<0.05), but decreased urinary nitrogen (UN) from 0.40 g/d to 0.29 g/d and 0.26 g/d (p<0.01) compared with the control group. As a result, the addition of L-Arg and NCG caused the retention nitrogen (RN) to increase from 2.39 g/d to 2.59 g/d and 2.60 g/d (p<0.05); net protein utilization ratio (NPU) to increase from 70.50% to 72.96% (p<0.01) and 73.65% (p<0.01); and biological value (BV) to increase from 85.66% to 89.93% and 90.91% (p<0.01), respectively.

Similar results were observed in the young rabbit stage. The addition of L-Arg and NCG in the diet increased IN from 4.05 g/d to 4.25 g/d and 4.24 g/d (p<0.01), increased FN from 0.74 g/d to 0.81 g/d and 0.82 g/d (p<0.05), and decreased UN from 0.52 g/d to 0.34 g/d and 0.31 g/d, respectively (p<0.01). NPU was also increased from 68.89% to 72.94% and 73.35% (p< 0.01). The BV was increased from 84.29% to 90.12% and 90.94% (p<0.01). No significant change in RN was observed at this stage. In addition, regardless of the growth stage, the addition of L-Arg and NCG had no significant effect on digestible nitrogen and apparent digestibility.

### Effects of L-arginine and N-carbamoylglutamic acid on Japanese White rabbits immune function

As shown in Table 4, in 65-day-old rabbits, the addition of L-Arg and NCG increased the IgM concentration from 0.053 g/L to 0.075 g/L and 0.063 g/L (p<0.01), respectively, and increased the IgG concentration from 1.006 g/L to 1.110 g/L and 1.089 g/L; the thymus index increased from 0.13 to 0.17 and 0.20, respectively (p<0.05), while L-Arg also increased the IgA concentration from 0.041 to 0.060 (p<0.01). However, NCG generated no such effect. L-Arg and NCG had no significant effect on the spleen index. In the 85-day-old rabbits, the addition of L-Arg and NCG increased the average IgA concentration from 0.046 g/L to 0.078 g/L and 0.074 g/L, respectively (p<0.01); the IgM concentration from 0.053 g/L to 0.077 g/L and 0.073 g/L (p<0.01); and the IgG concentration from 1.109 g/L to 1.358 g/L and 1.291 g/L (p<0.05). The thymus index and spleen index were not significantly affected (p>0.05).

### Effects of L-arginine and N-carbamoylglutamic acid on intestinal morphology in rabbits

As shown in Table 5, for the jejunum, the addition of L-Arg and NCG increased the VH of 65-day-old rabbits from 656.5 μm to 795.6 μm and 807.5 μm (p<0.05), respectively, and increased the CD from 67.8 μm to 78.0 μm and 81.5 μm (p< 0.05), respectively. However, the VH/CD (V/C) index showed no significant changes. For 85-day-old rabbits, the addition of L-Arg and NCG increased VH from 711.2 μm to 952.8 (p< 0.05) μm and 889.3 μm, respectively, with no significant effects on CD and the V/C index.

For the ileum, at 65 days of age, the addition of NCG increased the VH from 420.6 μm to 469.4 μm; the CD decreased from 77.2 μm to 54.6 μm, thus increasing the V/C index to 9.35 (p<0.05). At 85 days of age, L-Arg and NCG increased the ileal VH from 431.3 μm to 510.5 μm and 521.1 μm (p< 0.05), respectively, while the effect on V/C index was not significant.

### Illumina HiSeq-derived metadata

**Figure 2:**
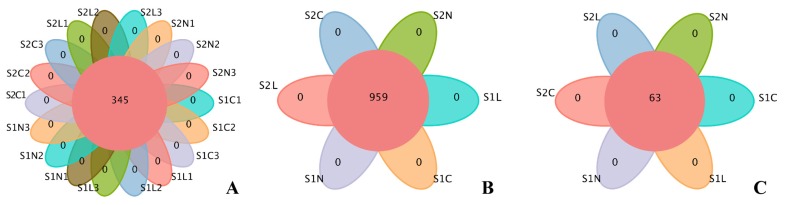
Flowerplots summarizing the numbers of common and unique OTUs. No special OTU among the 6 groups based on OTU and genus level analysis was identified. The numbers inside the diagram indicate the numbers of OTUs. Different colors represent different groups. (A) Numbers of common and unique OTUs in all samples based on the OTU level. (B) and (C) Numbers of common and unique OTUs and genera in all groups, respectively. OTUs, operational taxonomic units.

Overall, 18 samples of intestinal contents were used to assess the effects of L-Arg and NCG supplementation on cecal microflora of JWR. The sequencing quality is shown in Supplementary Table S1. On average, there were approximately 900 OTUs for each sample (Supplementary Figure S2) and more than 1,000 OTUs for every group (Supplementary Figure S3). No special OTU among the 6 groups based on OTU and genus level analysis was identified, as shown in Figure 2. The alpha diversity and rarefaction curves, Shannon index and rank abundance curve are shown in Supplementary Table S2 and Supplementary Figure S4 to S7. Bacterial alpha diversity showed no significant differences among all groups (p>0.05).

Subsequently, UPGMA-based Beta-sequence analysis and Metastats-based differences between groups showed that the proportion of microbial community composition in the control group, L-Arg-supplemented group and NCG-supplemented group changed significantly. The microbial community diversity change is shown in Table 6 and Supplementary Table S3.

In the first stage of feeding, the addition of L-Arg caused a significant decrease in *Parabacteroides* compared with the control group; the addition of NCG resulted in a significance decrease in the *Ruminococcaceae_V9D2013_group*, *Ruminococcaceae_UCG-005*, and *Parasutterella*, *Subdoligranulum group*, while *Ruminiclostridium_1* and *Roseburia* increased significantly. L-Arg and NCG supplementation led to the greatest change in the *Christensenellaceae_R-7_group*, *Gelria*, *Ruminococcaceae_UCG-001*, and *Enterorhabdus*.

In the second stage of feeding, compared with the control group, the continuous addition of L-Arg caused a significant decrease in *Clostridiales_bacterium* while *Anaerovorax* increased; the continuous addition of NCG significantly decreased *Alistipes* and *Bacteroides* while increasing the *Hydrogenoanaerobacterium*, *Tyzzerella_3*, and *[Eubacterium]_ruminantium_group*. The *Alistipes* and *Family_XIII_AD3011_group* have the largest changes in abundance differences between L-Arg and NCG. *Parasutterella*, *Barnesiella*, *Ruminococcaceae_V9D2013_group*, *Ruminiclostridium_1, dgA-11_gut_group*, *Ruminococcus_1*, *[Eubacterium]_ventriosum_group*, *Anaerrotruncus*, *[Eubacterium]_nodatum_group*, and *Hydrogenoanaerobacterium* showed the greatest changes between the control group and the L-Arg and NCG continuous addition groups between the different growth stages, respectively.

**Figure 3:**
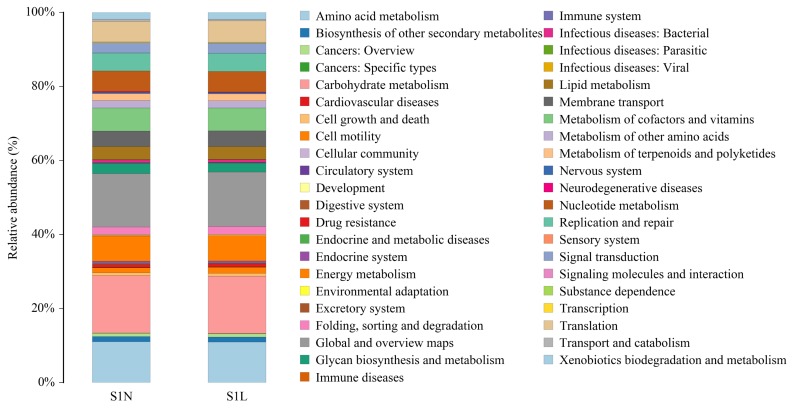
Relative abundance and biological function of the main bacterial communities in the S1N and S1L groups. Top 5 of the differential main functions of the species with an abundance value greater than 1% between S1N and S1L groups were carbohydrate metabolism, global and overview maps, amino acid metabolism, energy metabolism, and metabolism of cofactors and vitamins. The longitudinal coordinate represents the bacterial community relative abundance, with the same color labeling the associated biological function.

**Figure 4:**
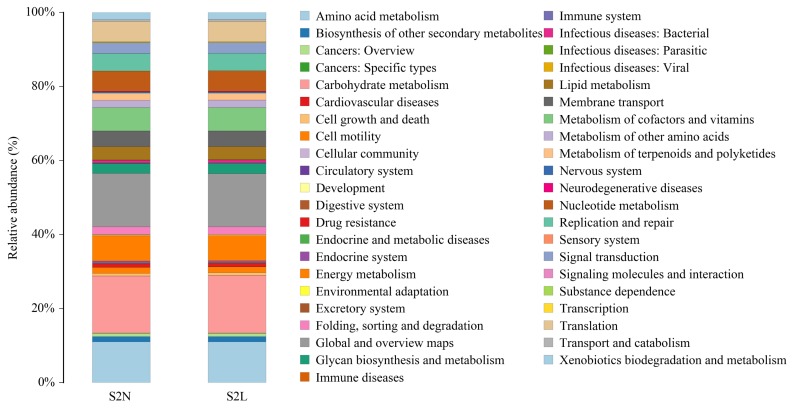
Relative abundance and biological function of the main bacterial communities in the S2N and S2L groups. Top 5 of the differential main functions of the species with an abundance value greater than 1% between S2N and S2L groups were carbohydrate metabolism, global and overview maps, amino acid metabolism, energy metabolism, and metabolism of cofactors and vitamins. The longitudinal coordinate represents the bacterial community relative abundance, with the same color labeling the associated biological function.

Based on the above results, we further analyzed the microbial community alteration-mediated gene functions and metabolic pathway changes caused by the addition of L-Arg and NCG in rabbits at different growth stages. The results showed that in the two growth stages (Figures 3, 4), the main functions of the species with an abundance value greater than 1% were carbohydrate metabolism, transcription, amino acid transport and metabolism, cell wall/membrane/envelope biogenesis, replication, recombination and repair, translation, ribosomal structure and biogenesis, signal transduction mechanisms, energy production and conversion, inorganic ion transport and metabolism, coenzyme transport and metabolism, defense mechanisms, posttranslational modification, protein turnover, chaperones, nucleotide transport and metabolism, lipid transport and metabolism, intracellular trafficking, secretion, and vesicular transport, cell cycle control, cell division, chromosome partitioning, cell motility, secondary metabolite biosynthesis, and transport and catabolism, as well as several unknown functions.

## DISCUSSION

As fur-producing animals and experimental animals, rabbits are particularly sensitive to a lack of essential amino acids, especially arginine [9]. An essential amino acid in many young animals, arginine has important nutritional, metabolic and immune functions and has an important impact on an animal’s reproductive ability. However, the effects and mechanism of arginine and its analogues on overall health and growth of rabbits, particularly its effects on the structure and proportions of the intestinal microbial community, are not clear. The addition of different feeds, antibiotics and additives has a significant impact on the microecological environmental balance in livestock and poultry. Therefore, in this study, we assessed the effects of NCG and L-Arg supplementation on rabbit growth, nitrogen metabolism, and immune capabilities. Effects of NCG and L-Arg supplementation on the intestinal microbial community were also analyzed by high-throughput sequencing techniques.

Feed intake is an important factor affecting animal production efficiency. It is also an important indicator for evaluating animal health and production performance. In this study, we found that daily weight gain and feed intake were significantly increased after adding L-Arg and NCG to the diet of weaned young rabbits. This may be because L-Arg and NCG can regulate the levels of hormones and endogenous nitric oxide in rabbits [10]. Moreover, feed intake is affected by gastrointestinal microbial community function. The differently altered microbiota in this study including *Clostridium*, *Ruminiclostridium*, and *Ruminococcaceae* have carbohydrate-active enzymes which actively degrades plants by sugar transport and metabolic pathways and could effectively utilize cellulose and raw lignocellulose feed-stocks to improve the digestibility of feed nutrition. They also increase the expression levels of insulin-like growth factor 1 and growth hormone receptor gene which enhance the growth of young animals effectively. These results are consistent with other studies that amino acid levels are critical for animal appetite and feed intake through their regulation effects on the of microbial community [11,12]. At the same time, we also found that both L-Arg and NCG were able to increase the daily weight gain of rabbits; NCG had a slightly greater effect on the intake of rabbits than L-Arg, but the difference was not significant. Addition of L-Arg and NCG did not have a significant impact on the feed conversion ratio. This was not consistent with previous reports that L-Arg and NCG can promote inconsistent feed conversion rates for pigs, cattle, and poultry. From alpha diversity calculation, we also found that there were no significant differences between groups. Ace and Chao1 indices reflect the OTU abundance in samples. Shannon and Simpson indices reflect the diversity of OTU in samples. The results indicate that L-Arg and NCG have no effect on the overall richness and evenness of microbiota. These may be because JWR are not used for meat production but rather function as experimental rabbits.

An immune organ is a tissue structure in which the body performs an immune function, and it is a place where lymphocytes and other immune cells cooccur, differentiate, proliferate, and generate an immune response. In general, it is believed that an increase in the immune organ index means that the animal’s immune system matures faster. The cytokines and antibodies with immune and defense functions are mostly composed of proteins. IgA, IgM, and IgG in the serum are important components of the immune system, which can effectively resist and reduce pathogenic bacteria. Protein and amino acid nutrition play an important role in maintaining the normal immune function of organisms. From our results, regardless of physiological stage, both the addition of L-Arg and NCG could increase the nitrogen metabolism, protein efficiency ratio, and BV. The urinary nitrogen level decreased significantly in the L-Arg and NCG-supplemented groups compared with the control group. In both stages of this study, L-Arg and NCG supplementation was effective in increasing IgM and IgG levels but had no effect on spleen index. In addition, L-Arg had a greater effect on increasing IgA levels in weanling rabbits than NCG. Both L-Arg and NCG could increase the Thymus Index in weanling rabbits. Therefore, the L-Arg and NCG could both significantly improve the nitrogen utilization rate and enhance immune function of JWR. These may first depend on the beneficial effects of L-Arg and NCG which are closely related to the nitrogen cycle and regulates the body’s nitrogen reserve. The microbiota including *Ruminococcus*, *Eubacterium*, *Hydrogenoanaerobacterium*, *Alistipes*, *Barnesiella*, and *Bacteroides* also have effects on regulation of short chain fatty acid production, increasing energy uptake efficiency, promoting vitamin synthesis, and further improving protein utilization [13,14]. At the same time, we noticed that *Ruminococcus*, *Ruminococcaceae*, *Alistipes*, *Parasutterella*, *Clostridia*, *Tyzzerella*, and *Enterorhabdus* had different relative abundance among different groups. These microbiota secrete volatile fatty acid which have been shown to have anti-inflammatory and immunity enhancement effects through farnesoid X receptor-induced antimicrobial peptides, interleukin-2 (IL-2), IL-4, IL-10, and immunoglobulins [15,16]. This may be the reason why supplementation of arginine and its analogues in diets can effectively increase thymus and spleen weight index and improve humoral immune function [17].

The normal structure and function of the small intestine provides the basic support for nutrients to be fully digested and absorbed. The VH is significantly correlated with the number of cells, and the number of cells determines the ability of the small intestine to absorb nutrients. The depth of the crypts reflects the rate of cell formation, and the shallower depth indicates an increase in the maturation rate of intestinal epithelial cells and an enhanced absorption function. The ratio of the two reflects the area of the intestinal lining, and a lower ratio indicates poorer intestinal digestive capacity [18]. Our results showed that L-Arg and NCG could significantly increase the VH and CD in the jejunum of weanling rabbits, which indicates that L-Arg and NCG can increase the intestinal absorption area but may be detrimental to intestinal epithelial cell maturation to some extent. However, there was no significant difference in V/C index, which indicates that there was no negative impact on the overall absorptive capacity of the intestines. For young rabbits, L-Arg and NCG could still increase the VH. In the ileum, NCG could significantly increase the VH and V/C index of weanling rabbits, reducing the CD; a similar effect was also observed in young rabbits. This indicates that L-Arg and NCG have beneficial promotional effects on rabbit small intestinal development, although NCG is more effective than L-Arg. The different microbial communities and abundance in different groups may be important factors which were affecting the growth and development of intestinal epithelial cells. The lithocholic acid, ursodeoxycholic acid, succinate and short-chain fatty acids (such as acetic, propionic, butyric, and valeric acid), which are regulated by *Anaerotruncus*, *Parabacteroides*, *Roseburia*, R*uminococcaceae*, *Subdoligranulum*, *Ruminococcus*, *Clostridiales bacterium*, provide energy, modulates host metabolism, repair intestinal barrier integrity and improve intestinal growth [19,20]. These microbiota also have effects on intestinal epithelial cell growth by regulating the gene expressions of insulin-like growth factor-binding protein 3, cyclin dependent kinase inhibitor 1A (*CDKN1A* or *p21*), and cyclin D1 [21].

Previous studies have shown that *Anaerovorax*, *Alistipes*, *Anaerotruncus*, *Bacteroides*, and other microbiotas with different abundance in different groups are related to feed conversion, immune function, incidence of inflammation, fatty acid utilization, microbiota recolonization, and environmental adaptability [22–27]. Combined with the results of COG analysis, we have shown that the main effects of L-Arg and NCG on the host include optimization of amino acid transport and metabolism, energy production and conversion, lipid transport and metabolism, and improvement of animal immunity. The difference between L-Arg and NCG is mainly in the promotion of nutrient utilization, relieving inflammation and enhancing adaptability.

The ileum is the last segment of the intestine where amino acids can be absorbed. However, the role of amino acids is not only to be a nutrient for the animal itself. The digestive bacteria can change the final bioavailability of the original amino acid by using the original amino acids derived from the digestive tract and its own endogenous amino acids [28]. The digestive physiology of rabbits is mainly based on the cecal microbial population, which is characterized by the presence of a rich microbial population. Furthermore, its composition and stability are of great significance in improving nutrient absorption and enhancing resistance. In this study, L-Arg and NCG supplementation changed the microbial communities and abundance. This may be because the addition of L-Arg and NCG affected the metabolism of the arginine-family of amino acids and the serine- and aspartate-family of amino acids in intestinal bacteria and reduced the utilization of most amino acids in mixed bacteria. Moreover, arginine and several other amino acids were largely utilized by the certain bacteria of intestines. L-Arg and NCG supplementation also improved the growth of cecal lactobacillus and anaerobic bacteria which plays important roles in maintaining gut health and functions, including balance of gut microflora [29,30]. Therefore, we could speculate that dietary L-Arg and NCG supplementation was beneficial for maintaining gastrointestinal function and health in young rabbits.

## CONCLUSION

In summary, this study preliminarily clarified the promotional ability of L-Arg and NCG on the growth and immunity of weanling and young JWR, as well as their effects on the jejunum and ileum villi. Furthermore, the effects of L-Arg and NCG on the structure and relative proportions of the cecal microbial community in these rabbits were clarified by high-throughput sequencing technology, which provides an exciting new avenue to further analyze the effects of the rabbit intestinal microbial community on nutrient absorption and utilization, the herbivorous processing mechanisms of rabbits, and the development of new feed additives.

## Supplementary Data





















## Figures and Tables

**Table 1:** Composition and nutrient levels of basal diet (air-dry basis, %)

Composition	Content	Nutrient composition	Nutrient levels1)
	
Corn	48.50	DE (MJ/kg)	10.45
Wheat bran	5.00	CP	15.70
Alfalfa	20.00	CF	10.95
Wildrye	10.00	EE	3.00
Corn oil	0.05	Met+Cys	0.70
Soybean meal	14.05	Lys	0.78
Calcium hydrophosphate	0.50	Calcium	1.00
Limestone	0.90	Phosphorus	0.50
Premix2)	1.00	Arginine	0.77
Total	100	Glutamic acid	0.72

DE, digestible energy; CP, crude protein; CF, crude fiber; EE, ether extract; Met+Cys, methionine and cysteine; Lys, lysine.

1) Nutrient levels represent calculated values.

2) The premix contains in the following per kg: vitamin A, 12,000 IU; vitamin D_3_, 1,000 IU; vitamin E, 50 mg; iron, 50 mg; copper, 5 mg; manganese, 30 mg; zinc, 50 mg; selenium, 0.08 mg; iodine, 0.1 mg; and cobalt, 0.2 mg.

**Table 2:** Effects of L-arginine and N-carbamoylglutamic acid on growth performances of Japanese White rabbits

Stage	Inspection	Control	L-arginine	N-carbamoylglutamic acid
Day 37–65 (Stage 1)	ADG (g)	37.38±1.57^A^	39.40±1.28^B^	39.53±1.35^B^
	ADFI (g)	100.05±0.44^a^	101.14±0.68^b^	101.70±1.06^b^
	FCR (%)	37.36±1.24	38.92±0.22	39.08±0.62
Day 66–85 (Stage 2)	ADG (g)	37.16±0.99^A^	39.15±1.00^B^	39.68±1.98^B^
	ADFI (g)	125.46±0.79^A^	128.75±1.49^B^	131.33±2.18^B^
	FCR (%)	29.62±0.72	30.41±1.03	30.21±0.31

ADG, average daily gain; ADFI, average daily feed intake; FCR, feed conversion rate.

Significant differences in the same line are represented with different lower-case letters (p<0.05) and different capital letters (p<0.01).

**Table 3:** Effects of L-arginine and N-carbamoylglutamic acid on metabolic nitrogen levels of Japanese White rabbits

Stage	Test items	Control	L-arginine	N-carbamoylglutamic acid
Day 63 to 65	IN (g/d)	3.39±0.03^A^	3.55±0.03^B^	3.53±0.04^B^
	FN (g/d)	0.60±0.03^a^	0.67±0.03^b^	0.67±0.02^b^
	UN (g/d)	0.40±0.15^A^	0.29±0.02^B^	0.26±0.02^B^
	DN (g/d)	2.79±0.05	2.88±0.05	2.86±0.02
	RN (g/d)	2.39±0.04^a^	2.59±0.04^b^	2.60±0.02^b^
	AD (%)	82.30±0.80	81.13±1.08	81.02±1.31
	NPU (%)	70.50±0.54^A^	72.96±0.85^B^	73.65±1.02^B^
	BV (%)	85.66±0.57^A^	89.93±0.67^B^	90.91±0.51^B^
Day 83 to 85	IN (g/d)	4.05±0.02^A^	4.25±0.02^B^	4.24±0.03^B^
	FN (g/d)	0.74±0.04^a^	0.81±0.05^b^	0.82±0.02^b^
	UN (g/d)	0.52±0.02^A^	0.34±0.02^B^	0.31±0.02^B^
	DN (g/d)	3.31±0.03	3.44±0.03	3.42±0.03
	RN (g/d)	2.79±0.01	3.1±0.01	3.11±0.04
	AD (%)	81.73±0.53	80.94±0.38	80.66±0.93
	NPU (%)	68.89±0.16^A^	72.94±0.54^B^	73.35±1.15^B^
	BV (%)	84.29±0.39^A^	90.12±1.03^B^	90.94±0.49^B^

IN, intake nitrogen; FN, fecal nitrogen; UN, urinary nitrogen; DN, digestible nitrogen; RN, retention nitrogen; AD, apparent digestibility; NPU, net protein utilization; BV, biological value.

Significant differences in the same line are represented with different lower-case letters (p<0.05) and different capital letters (p<0.01).

**Table 4:** Effects of L-arginine and N-carbamoylglutamic acid on improvement of Japanese White rabbits immunity

Test items	Control	L-arginine	N-carbamoylglutamic acid
Day 65			
IgA (g/L)	0.041±0.004^A^	0.060±0.002^B^	0.046±0.004^A^
IgM (g/L)	0.053±0.003^A^	0.075±0.002^B^	0.063±0.005^B^
IgG (g/L)	1.006±0.083^a^	1.110±0.024^b^	1.089±0.024^b^
Thymus index	0.13±0.02^a^	0.17±0.04^b^	0.20±0.03b
Spleen index	0.06±0.01	0.06±0.02	0.06±0.01
Day 85			
IgA (g/L)	0.046±0.005^A^	0.078±0.006^B^	0.074±0.004^B^
IgM (g/L)	0.053±0.006^A^	0.077±0.008^B^	0.073±0.004^B^
IgG (g/L)	1.109±0.034^a^	1.358±0.179^b^	1.291±0.051^b^
Thymus index	0.14±0.18	0.14±0.13	0.14±0.20
Spleen index	0.06±0.01	0.06±0.01	0.06±0.01

Ig, immunoglobulin.

Thymus and spleen index were expressed as the thymus or spleen weight relative as body weight.

Significant differences in the same line are represented with different lower-case letters (p<0.05) and different capital letters (p<0.01).

**Table 5:** Effects of L-arginine and N-carbamoylglutamic acid in development of Japanese White rabbits ileum and jejunum tissue

Age	Test items	Control	L-arginine	N-carbamoylglutamic acid
Jejunum				
Day 65	VH	656.5±63.1^a^	795.6±90.7^b^	807.5±71.5^b^
	CD	67.8±13.6 ^a^	78.0±21.5^b^	81.5±27.5^b^
	V/C index	10.1±2.3	10.8±2.8	10.3±4.6
Day 85	VH	711.2±124.9^a^	952.8±162.5^b^	889.3±101.9^ab^
	CD	85.3±19.9	88.7±21.0	96.1±31.1
	V/C index	9.3±2.7	11.1±2.2	10.1±3.2
Ileum				
Day 65	VH	420.6±53.4^a^	456.8±62.7^ab^	469.4±81.7^b^
	CD	77.2±15.9^a^	72.2±21.4^a^	54.6±16.6^b^
	V/C index	5.7±1.7^a^	6.7±1.5^a^	9.35±3.4^b^
Day 85	VH	431.3±88.1^a^	510.5±70.5^b^	521.1±47.9^b^
	CD	90.2±23.5^a^	75.1±18.8^b^	78.3±21.5^b^
	V/C index	5.0±1.5	7.3±2.5	7.6±1.5

VH, villus height (μm); CD, crypt depth (μm); V/C index, villus height/crypt depth.

Significant differences in the same line are represented with different lower-case letters (p<0.05).

**Table 6:** Microbial community composition changing based on the Metastats analysis

Genus	S1C vs S1L	S1C vs S1N	S1L vs S1N	S2C vs S2L	S2C vs S2N	S2L vs S2N	S1C vs S2C	S1L vs S2L	S1N vs S2N
*Parabacteroides*	4.08								
*Ruminococcaceae_V9D2013_group*		2.49					0.62		
*Ruminiclostridium_1*		0.43					3.01		
*Ruminococcaceae_UCG-005*		3.45							
*Parasutterella*		2.18							
*Roseburia*		0.18							
*Subdoligranulum*		1.74							
*Parasutterella*							0.32		
*Barnesiella*							3.34		
*dgA-11_gut_group*							21.44		
*Ruminococcus_1*							0.70		1.30
*Christensenellaceae_R-7_group*			1.74						
*Gelria*			-						
*Ruminococcaceae_UCG-001*			-						
*Enterorhabdus*			0.40						
*[Eubacterium]_ventriosum_group*								0.44	
*Anaerotruncus*								0.53	
*[Eubacterium]_nodatum_group*								0.13	
*Hydrogenoanaerobacterium*									0.34
*Clostridiales_bacterium*				4.97					
*Anaerovorax*				0.49					
*Alistipes*					3.08	2.73			
*Hydrogenoanaerobacterium*					-				
*Tyzzerella_3*					0.41				
*Bacteroides*					2.55				
*[Eubacterium]_ruminantium_group*					0.06				
*Family_XIII_AD3011_group*						1.41			

The values represent the relative abundance ratios of the modified species in the two groups.

“−” represent the modified species is not detectable in one of those two groups.
