# Supplementary material for: L-arginine and N-carbamoylglutamic acid supplementation enhance young rabbit growth and immunity by regulating intestinal microbial community
Source: Asian-Australas J Anim Sci. 2019 May 28;33(1):166–76. doi: 10.5713/ajas.18.0984 (PMC6946986; doi:10.5713/ajas.18.0984)
Supplement: Supplementary file 1 [file ajas-18-0984-suppl1.pdf]

Supplementary Table S1. Sequencing data quality assessment

Sample ID	PE Reads	Raw Tags	Clean Tags	Effective Tags	AvgLen (bp)	GC (%)	Q20 (%)	Q30 (%)	Effective (%)
S1C1	79,612	65,994	55,764	55,754	412	52.95	94.31	88.15	70.03
S1C2	79,764	66,872	56,704	56,587	413	53.44	94.28	88.10	70.94
S1C3	79,794	69,039	60,461	60,415	412	52.67	94.79	89.17	75.71
S1L1	79,936	66,618	55,891	55,785	414	54.02	94.42	88.28	69.79
S1L2	79,930	67,031	57,483	57,300	411	53.60	94.38	88.24	71.69
S1L3	80,078	67,146	57,512	57,214	411	53.84	94.35	88.19	71.45
S1N1	80,043	67,007	57,646	57,511	413	52.98	94.66	88.90	71.85
S1N2	79,921	66,687	56,772	56,642	413	53.53	94.62	88.71	70.87
S1N3	80,338	66,982	57,334	57,203	414	53.79	94.73	88.99	71.2
S2C1	79,932	68,990	60,181	59,953	412	53.54	94.99	89.52	75.01
S2C2	80,448	67,474	58,186	58,091	411	53.08	94.42	88.36	72.21
S2C3	80,024	68,193	59,081	59,071	413	52.65	94.68	88.94	73.82
S2L1	79,759	67,933	59,656	59,599	409	52.63	94.74	89.05	74.72
S2L2	80,307	69,047	60,148	60,078	413	52.55	94.69	88.97	74.81
S2L3	80,134	66,939	57,065	56,977	413	53.81	94.51	88.50	71.1
S2N1	79,985	67,142	57,358	57,288	413	53.39	94.58	88.70	71.62
S2N2	80,006	67,727	58,602	58,554	413	52.71	94.57	88.74	73.19
S2N3	80,203	66,981	57,617	57,410	411	53.31	94.42	88.33	71.58
