# Supplementary material for: L-arginine and N-carbamoylglutamic acid supplementation enhance young rabbit growth and immunity by regulating intestinal microbial community
Source: Asian-Australas J Anim Sci. 2019 May 28;33(1):166–76. doi: 10.5713/ajas.18.0984 (PMC6946986; doi:10.5713/ajas.18.0984)
Supplement: Supplementary file 2 [file ajas-18-0984-suppl2.pdf]

Supplementary Table S2 Alpha diversity (Chao1、Ace、Shannon、Simpson) of sequenced samples

Sample ID	OTU	ACE	Chao1	Simpson	Shannon	Coverage
S1C1	896	949.741	963.8333	0.0238	4.9934	0.9964
S1C2	884	963.9462	967.1786	0.0232	4.8701	0.9956
S1C3	921	956.3959	955.5149	0.0231	5.0183	0.9976
S1L1	890	932.3406	937.5	0.0313	4.7803	0.9969
S1L2	964	1,008.6937	1,018.7979	0.0171	5.1741	0.9967
S1L3	868	922.9498	935.6552	0.0172	5.0837	0.9963
S1N1	943	996.4895	1,012.2581	0.0158	5.0858	0.9965
S1N2	869	977.0273	987.1651	0.0448	4.4667	0.995
S1N3	912	993.7765	1,002.5505	0.0253	4.8933	0.9957
S2C1	949	1,016.6593	1,024.0545	0.0228	4.94	0.9963
S2C2	922	970.9911	971.6364	0.0233	4.9925	0.9967
S2C3	920	969.5204	983.7766	0.0184	4.9961	0.9968
S2L1	921	977.8398	993.033	0.03	4.8099	0.9967
S2L2	917	975.5155	983.8667	0.0231	4.9449	0.9965
S2L3	864	914.0433	920.4839	0.0177	5.0409	0.9968
S2N1	909	954.435	953.5946	0.0167	5.0316	0.9969
S2N2	917	971.6473	983.6105	0.0187	5.0215	0.9966
S2N3	934	987.6028	999.2371	0.0255	4.995	0.9964
