# Supplementary material for: L-arginine and N-carbamoylglutamic acid supplementation enhance young rabbit growth and immunity by regulating intestinal microbial community
Source: Asian-Australas J Anim Sci. 2019 May 28;33(1):166–76. doi: 10.5713/ajas.18.0984 (PMC6946986; doi:10.5713/ajas.18.0984)
Supplement: Supplementary file 3 [file ajas-18-0984-suppl3.pdf]

Supplementary Table S3. Microbial communities and abundance changed in different groups

genus	S1C	S1L	S1N	S2C	S2L	S2N
Parabacteroides	0.14±0.0371	0.0343±0.0197				
Ruminococcaceae_V9D2013_group	2.13±0.064		0.856±0.243			
Ruminiclostridium_1	0.0328±0.00417		0.0769±0.0126			
Ruminococcaceae_UCG-005	0.442±0.0993		0.128±0.0532			
Parasutterella	0.993±0.0801		0.456±0.192			
Roseburia	0.00612±0.00159		0.0338±0.0108			
Subdoligranulum	0.405±0.0645		0.233±0.0221			
Parasutterella	0.315±0.0244			0.993±0.0801		
Barnesiella	0.212±0.0259			0.0635±0.0321		
Ruminococcaceae_V9D2013_group	1.32±0.235			2.13±0.064		
Ruminiclostridium_1	0.0987±0.0228			0.0328±0.00417		
dgA-11_gut_group	2.53±0.964			0.118±0.118		
Ruminococcus_1	11.5±1.7			16.4±1.05		
Christensenellaceae_R-7_group		12.1±1.52	6.94±0.547			
Gelria		0.0158±0.0052	0±0			
Ruminococcaceae_UCG-001		0.0114±0.0041	0±0			
Enterorhabdus		0.00939±0.00375	0.0236±0.00374			
[Eubacterium]_ventriosum_group		0.00736±0.00121			0.0168±0.000546	
Anaerotruncus		0.339±0.0961			0.643±0.0228	
[Eubacterium]_nodatum_group		0.0113±0.00406			0.0852±0.0275	
Ruminococcus_1			16.3±0.31			12.5±0.12
Hydrogenoanaerobacterium			0.00412±0.00107			0.0122±0.00306
Clostridiales_bacterium				0.197±0.039	0.0396±0.0122	
Anaerovorax				0.0762±0.0104	0.154±0.0197	
Alistipes				3.54±0.19		1.15±0.0776
Hydrogenoanaerobacterium				0±0		0.0122±0.00306
Tyzzerella_3				0.0536±0.00711		0.132±0.0225
Bacteroides				3.34±0.309		1.31±0.597
[Eubacterium]_ruminantium_group				0.0102±0.00368		0.168±0.0594
Alistipes					3.14±0.162	1.15±0.0776
Family_XIII_AD3011_group					0.0717±0.00552	0.0508±0.00585
