# Supplementary material for: L-arginine and N-carbamoylglutamic acid supplementation enhance young rabbit growth and immunity by regulating intestinal microbial community
Source: Asian-Australas J Anim Sci. 2019 May 28;33(1):166–76. doi: 10.5713/ajas.18.0984 (PMC6946986; doi:10.5713/ajas.18.0984)
Supplement: Supplementary file 4 [file ajas-18-0984-suppl4.pdf]

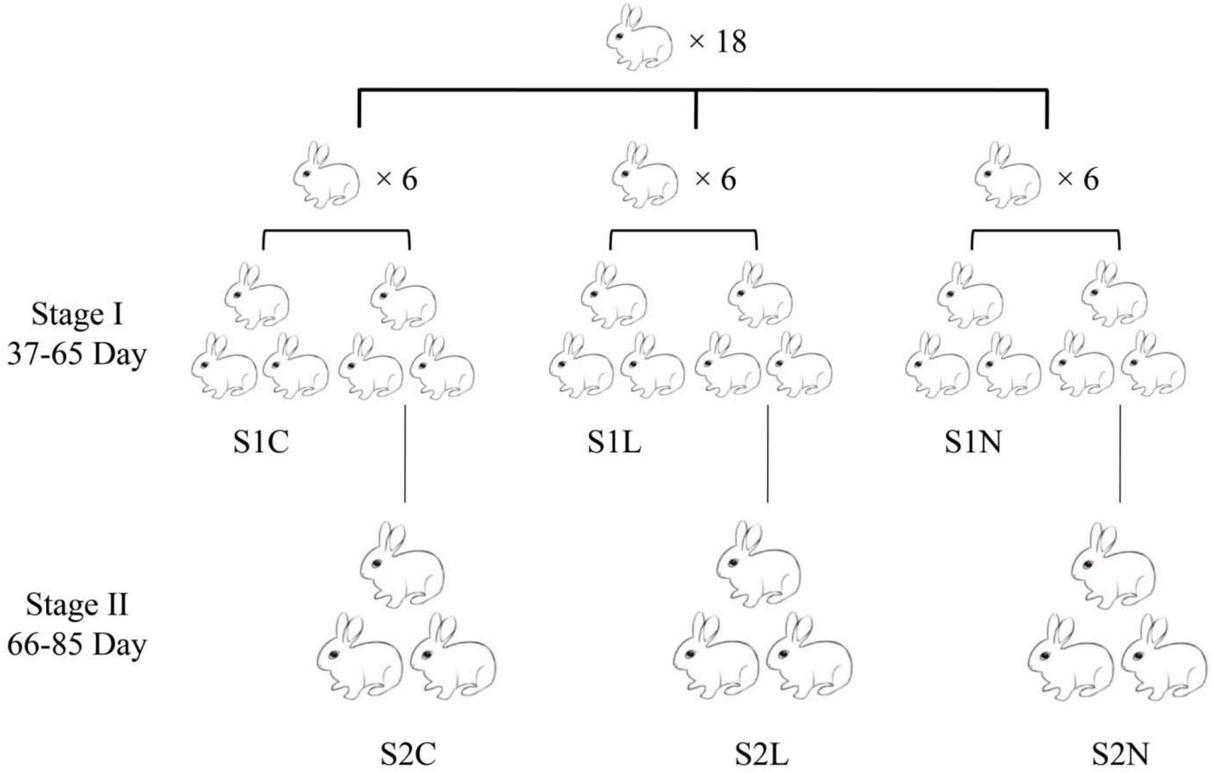

Supplementary Figure S1. The experimental scheme.

A total of 18 experimental rabbits of the same age and similar physiological status were randomly divided into 3 groups. According to the experimental design, different diets with no additives, L-Arg supplementation and NCG supplementation were added, respectively. After reaching the corresponding age, three rabbits in each group were randomly selected and used as the experimental groups S1C, S1L and S1N to perform the corresponding experiments. The remaining rabbits were maintained according to the original diet formula. After reaching the corresponding age, they were used to perform the corresponding experimental studies as S2C, S2L and S2N.
