# Supplementary material for: L-arginine and N-carbamoylglutamic acid supplementation enhance young rabbit growth and immunity by regulating intestinal microbial community
Source: Asian-Australas J Anim Sci. 2019 May 28;33(1):166–76. doi: 10.5713/ajas.18.0984 (PMC6946986; doi:10.5713/ajas.18.0984)
Supplement: Supplementary file 6 [file ajas-18-0984-suppl6.pdf]

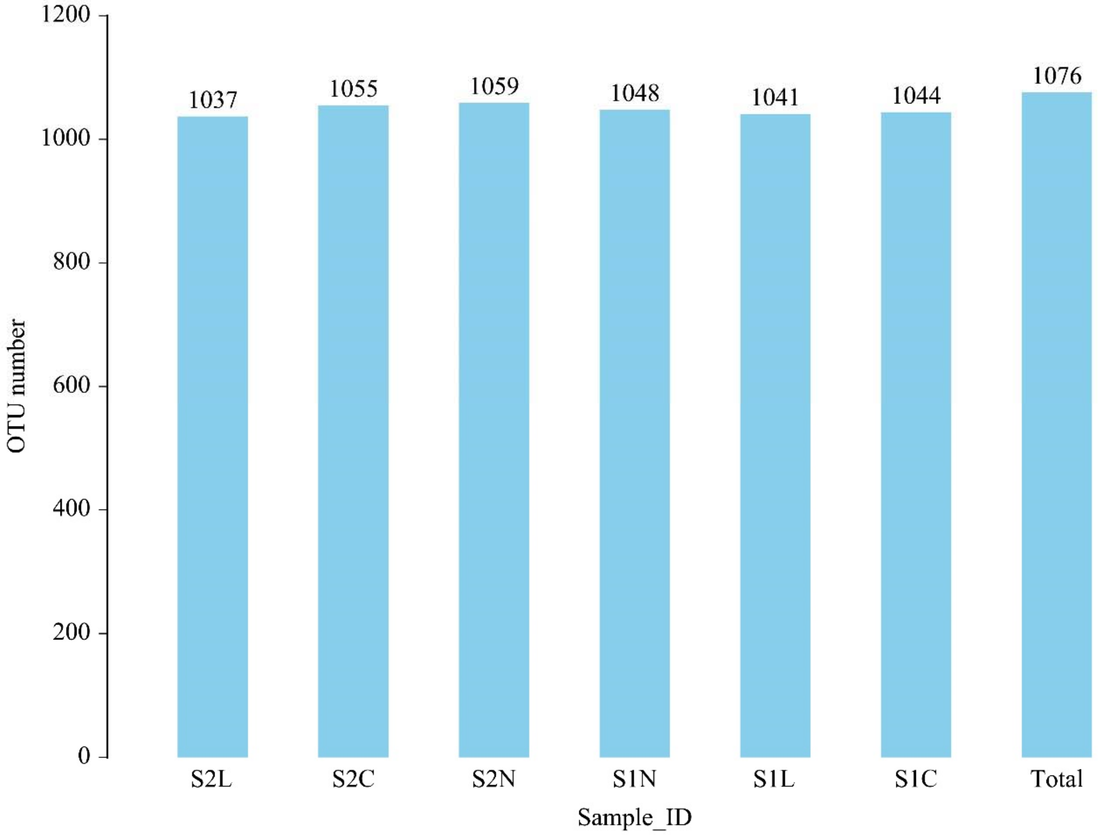

Supplementary Figure S3. Numbers of OTUs detected in all groups.

The horizontal coordinate represents the group name, while the longitudinal coordinate indicates their detected OTUs.
