# Supplementary figures and images for: L-arginine and N-carbamoylglutamic acid supplementation enhance young rabbit growth and immunity by regulating intestinal microbial community
Source: Asian-Australas J Anim Sci. 2019 May 28;33(1):166–76. doi: 10.5713/ajas.18.0984 (PMC6946986; doi:10.5713/ajas.18.0984)

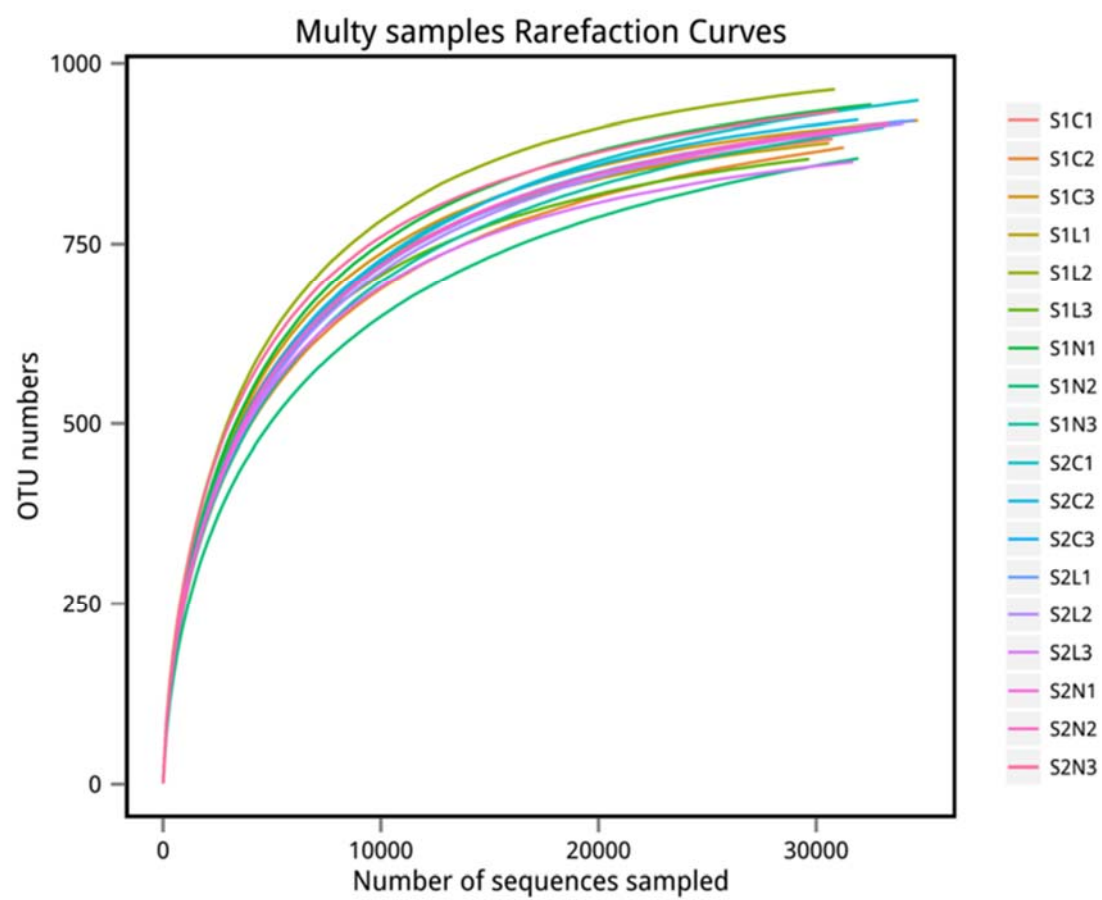

**Supplementary Figure S4.** Rarefaction curves for all samples.

Supplement: Supplementary file 7 [file ajas-18-0984-suppl7.pdf]

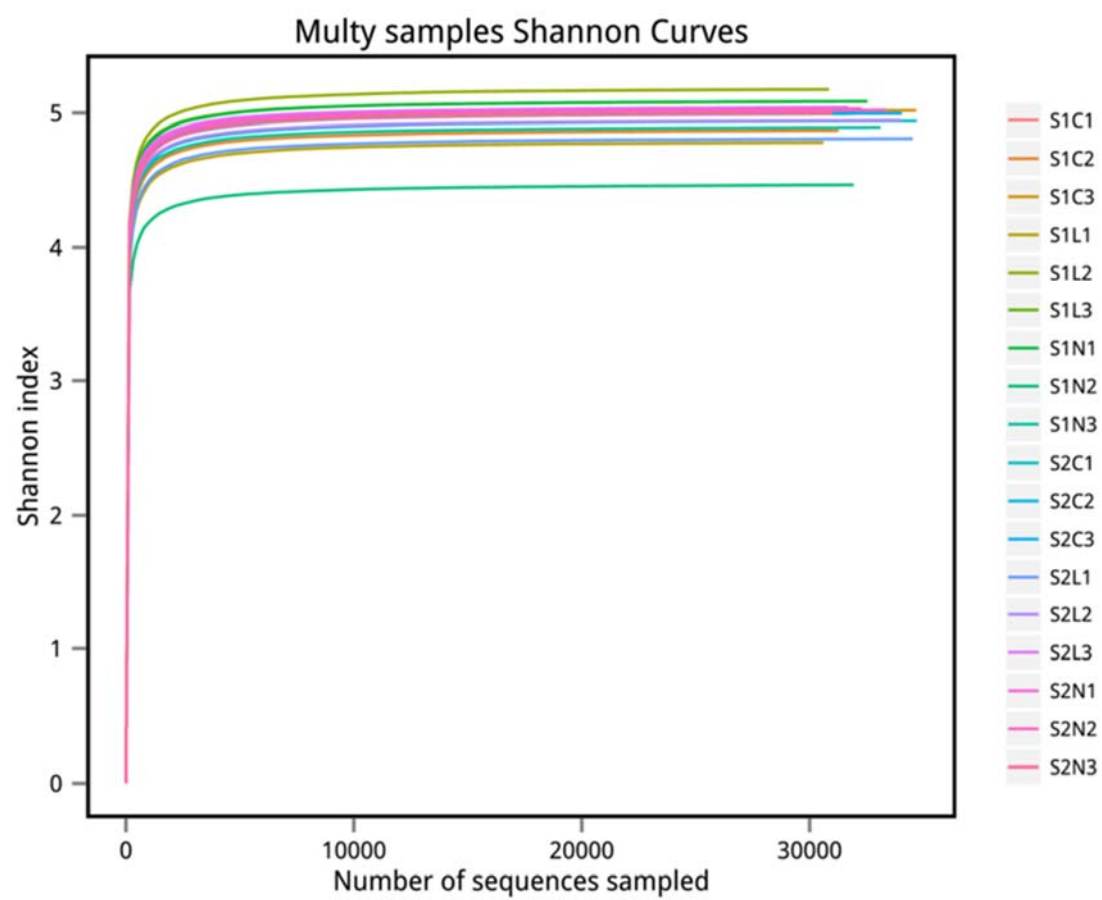

**Supplementary Figure S5.** Shannon curves for all samples.

Supplement: Supplementary file 8 [file ajas-18-0984-suppl8.pdf]

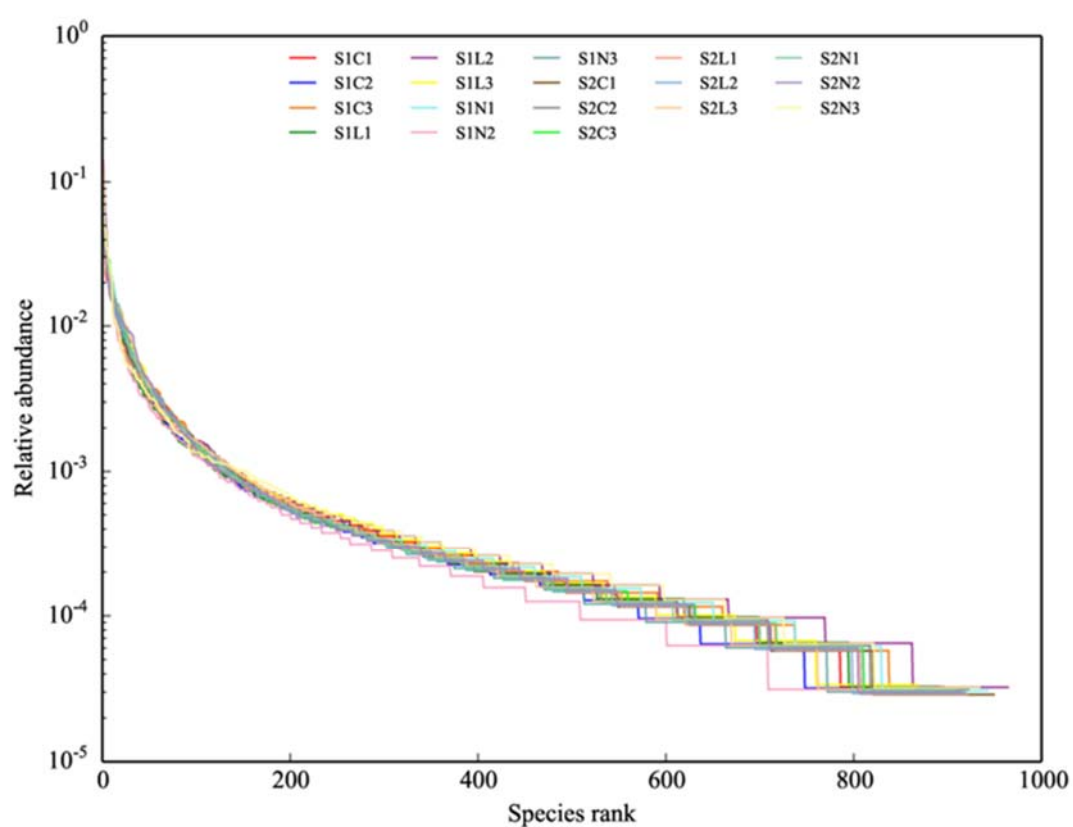

**Supplementary Figure S6.** Rank Abundance curve for all samples.

Supplement: Supplementary file 9 [file ajas-18-0984-suppl9.pdf]
