# Supplementary material for: L-arginine and N-carbamoylglutamic acid supplementation enhance young rabbit growth and immunity by regulating intestinal microbial community
Source: Asian-Australas J Anim Sci. 2019 May 28;33(1):166–76. doi: 10.5713/ajas.18.0984 (PMC6946986; doi:10.5713/ajas.18.0984)
Supplement: Supplementary file 10 [file ajas-18-0984-suppl10.pdf]

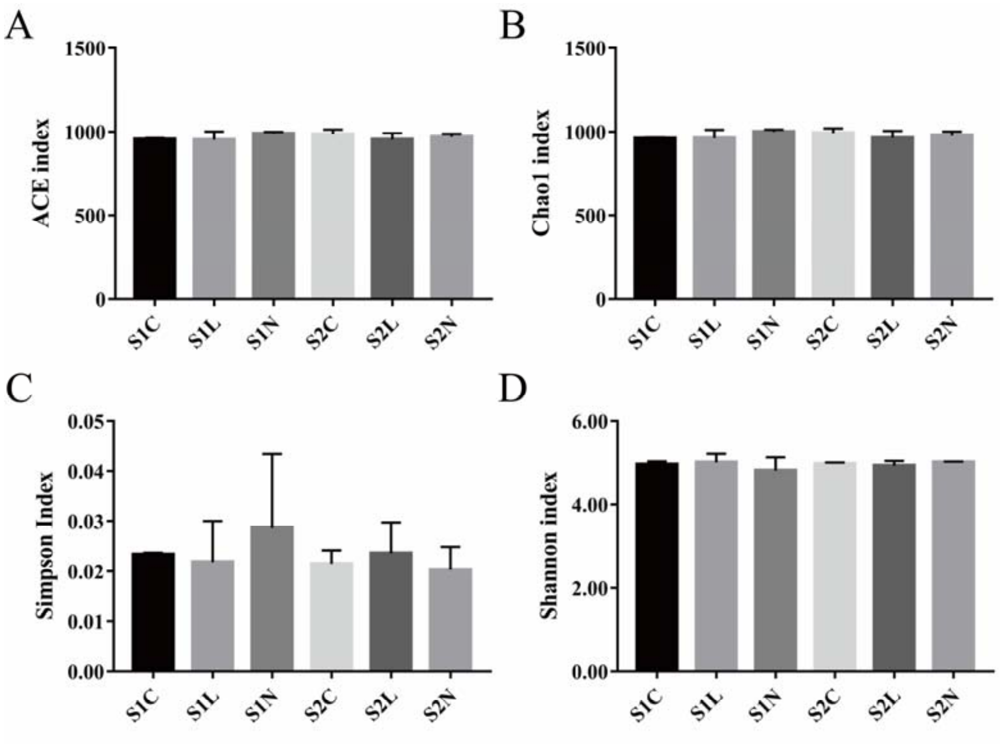

Supplementary Figure S7. Variations in alpha diversity of all groups.

Comparisons of the bacterial community ACE (A), Chao1 (B) Simpson (C), and Shannon (D) indexes within the different groups.
